# COVID-19 in Yemen: a crisis within crises

**DOI:** 10.1186/s12939-020-01231-2

**Published:** 2020-07-11

**Authors:** Seyyed Meysam Mousavi, Mina Anjomshoa

**Affiliations:** Department of Health Management and Economics, School of Public Health, Tehran University of Medical Sciences, Tehran, Iran

**Keywords:** COVID-19, Yemen, Crisis, War

## Abstract

Yemen is suffering deadly airstrikes and heavy bombardment since March 2015 which has created one of the most severe humanitarian crises worldwide. In this miserable situation, several communicable diseases have massively re-emerged including cholera, diarrhea, dengue, and measles, as a result of weapons used during the years of war according to geospatial patterns of the infected cases. According to the world health organization (WHO), only 51% of health care facilities across the country are fully functional, mainly due to the war. The fragile health system has extremely limited capacity to adopt and implement effective preparedness and response measures to the COVID-19 outbreak. The first and most imperative step to combat COVID-19 in Yemen is ending the devastating war without delay and terminating the land, sea and air blockade imposed by the coalition. International humanitarian organizations should also dedicate a high level joint action to implement a series of well-coordinated measures emphasizing both whole-of-government and whole-of-society approach to protect Yemenis’ right in life and health.

## Background

COVID-19 stands for coronavirus disease 2019 which still continues to spread rapidly and affects people across the globe. As the last country involved, Yemen has reported the first confirmed case for COVID-19 in Hadhramaut, the southern province, in the 10th of April 2020 [1]. The growing number of COVID-19 positive cases in neighboring countries surrounding Yemen [2] such as Saudi Arabia, Oman, Eritrea, and Somalia, indicates the likelihood of more positive cases but undetected so far. Considered the underlying causes, the unstable situation resulting from war and fragile health system have led to low capacity for testing in terms of logistics and sites, as only three sites are available in the whole country. In Yemen, the main concern is the proliferation of COVID-19 which might take place rapidly and lead to higher mortality rates if compared with the global average, and this can be attributed to several reasons. Nowadays, Yemen is deemed as the poorest country in the Middle East and North Africa, in addition, Yemenis suffer a fierce war which exposed them to uncountable deadly airstrikes and heavy bombardment since March 2015 [3, 4]. Accordingly, this has led to creating an unprecedented humanitarian crises, as more than 100 thousands people have been killed - a quarter of them are from women and children, more than 3.6 million people have been migrated forcefully, 24.3 million are in a pressing need of humanitarian aids for survival where Yemeni people have experienced food insecurity in more than 230 out of 333 governorates [5].

## Main text

In this wretched situation, several communicable diseases have massively re-emerged including, but not limited to, cholera, diarrhea, dengue, and measles as - at best indirect and at worst direct - weapons used during the years of war according to geospatial patterns of the infected cases [4]. As an example, more than 2.3 million suspected cases of cholera have been reported since the beginning of the outbreak in 2017 – as the worst documented epidemic in the history [6, 7]. The unsafe shelters, persistent migration and displacement, lack of essential medicines, inadequate food and insufficient access to safe water for drinking, suppressed immunity among malnourished population, and lacking the lower limit of hygiene standards would put Yemen at a high risk of emergence of severe outbreaks such as COVID-19 [8], but, with more dangerous implications than any elsewhere.

Awfully, the most recent update from the WHO mentioned that only 50% of health care facilities across the country are still able to work, while, the rest has been fully destroyed by the coalition airstrikes indiscriminately or deliberately in some occasions [4, 9]. Currently, this gradual collapse in the health system produces severe shortages in essential medicines, staff and equipment, as 700 intensive care unit beds are only available to cover all population needs [1], and basic healthcare services are inaccessible to three quarters of Yemenis [10]. This obviously neglected system does have extremely limited capacity for effective resilience including response measures to the COVID-19 outbreak, this pandemic which threatens the strong health systems in high-income countries as well as other countries around the world.

Based on latest estimation of the Ministry of Public Health and Population in Yemen, COVID-19 might expand and potentially infect 90% of Yemenis [11]. This serious estimations will necessitate the reallocation of those already scarce resources towards the new threat allowing those previously existing outbreaks to spread and become out of control. In summation, these factors together are expected to create severe and life-threatening circumstances which might leave a huge number of deaths among Yemenis, even more than the daily airstrikes do.

## Conclusions

Such letter is like an urgent humanitarian call to take the international responsibility towards establishing an action agenda in Yemen including emergency measures and scaling up preparedness in order to possess the relative capacity required for fighting against the COVID-19 pandemic. The first and most imperative step is to end the devastating war without delay and terminate the land, sea and air blockade imposed by the Saudi-UAE-led coalition that is because health is totally bound to security. In 23rd March 2020, the secretary general of the United Nations (UN) has strictly called for a global cessation of hostilities and end the airstrikes in all warring parties in response to the COVID-19 pandemic [12]. Accordingly, the coalition announced a two-week comprehensive ceasefire in 8th April 2020, however, it lasted just for 1 day where the coalition’s warplanes struck different civilian targets in various regions in Sa’ada, Amran, and al-Bayda [13]. Therefore, global health community has a paramount role in terminating this blockade and enduring fierce war for allowing the crippled health system to recover and perform its role in providing services to these millions of vulnerable inhabitants. Furthermore, the UN Security Council is invited to commit its role responsibly in banning transactions of armed weapons with the coalition especially from countries which are permanent members in the council [14], as an evidence showing the use of these weapons against civic targets, particularly healthcare infrastructure [4]. If this man-made war persists, it will trigger the dire starvation and aggravate the effects of current outbreaks that are almost impossible to address by global efforts. Such crisis not only poses a substantial threat to Yemen’s nationwide population, but also threatens the entire world to experience an endless COVID-19 pandemic.

Cessation of the war could be an important step, but alone will not definitely pause the proliferation of COVID-19 in the country. Therefore, international humanitarian organizations should also dedicate a high level joint action to implement a series of well-coordinated measures emphasizing both whole-of-government and whole-of-society approach to protect Yemenis’ right in life and health. The international organizations should also reinforce the Yemeni health system through, but not limited to; improving laboratory capacity to be capable of preventing the pandemic spread, providing life-saving pharmaceuticals and medical disposables, training health care workers, enhancing surveillance of entry-points, providing personal protection equipment, and establishing well-equipped centers for isolation and quarantine to fit the risky environments in the camps and illegal slums. Multi-sectoral movement with assertive commitment is also required to address these shortcomings and appropriately respond to the aforementioned demands to accomplish the comprehensive measures and rebuild infrastructures such as health care facilities, proper supplies of water and electricity, and hygiene sanitation.

## Data Availability

Not applicable.

## Abbreviations

COVID-19: Coronavirus disease 2019
the UN: The United Nations
WHO: World health organization
